# Using Olive Leaves to Enrich Olive Oil With Oleuropein Aglycone

**DOI:** 10.1002/fsn3.72421

**Published:** 2026-10-23

**Authors:** Jade te Bogt, David Speed, Ralf C. Schlothauer, Jed W. Fahey, Megan N. C. Grainger

**Affiliations:** ^1^ School of Science, Division of STEM The University of Waikato Hamilton New Zealand; ^2^ ALS Food & Environmental NZ Formerly Analytica Laboratories Hamilton New Zealand; ^3^ Whio Innovations Ltd Tauranga New Zealand; ^4^ Department of Medicine, Physiology, Pharmacology & Therapeutics, Psychiatry & Behavioral Sciences, and iMIND Hopkins, School of Medicine Johns Hopkins University Baltimore Maryland USA; ^5^ Institute of Medicine University of Maine Orono Maine USA

**Keywords:** β‐glucosidase, almond, enzymatic hydrolysis, extraction, oil enrichment, oleuropein, oleuropein aglycone, olive oil

## Abstract

Oleuropein is present in olive fruit and is converted during olive‐oil manufacture into oleuropein aglycone, other downstream secoiridoid derivatives, and phenolic alcohols. Oleuropein aglycone is among the major phenolic constituents of extra virgin olive oil; however, concentrations of these compounds can vary substantially between batches of oil. Olive leaves, an oleuropein‐rich by‐product of the olive industry, may provide a source of phenolic compounds for targeted olive‐oil enrichment. This study investigated the prospect of extracting phenolic compounds, particularly oleuropein, from olive leaves into an aqueous extract followed by enzymatic hydrolysis to oleuropein aglycone and assessment of its transfer to an oil phase. The impact of parameters such as dryness of the leaves when making olive leaf aqueous extract and different sources of β‐glucosidase (almond flour, almond meal, and almond milk) to hydrolyse oleuropein to the aglycone were investigated. Among the conditions tested, drying leaves at 70°C produced the highest oleuropein concentration in the extract, and almond milk as the source of enzyme was the best combination to achieve hydrolysis of oleuropein. However, the transfer of the oleuropein aglycone from the aqueous extract to the oil phase was inefficient under the conditions tested, indicating that multiple factors were limiting the mass transfer.

## Graphical abstract

Oleuropein was extracted from olive leaf by‐products and enzymatically converted to the bioactive oleuropein aglycone. Although hydrolysis was successful, oil enrichment was limited (6.5 mg/kg), likely due to micelle formation and complex aqueous–oil interfacial interactions, highlighting the need for further optimisation of oil enrichment strategies.

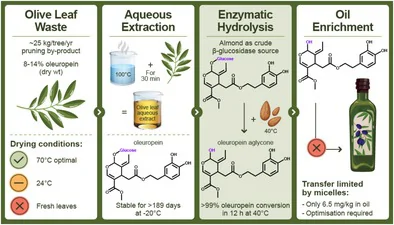

## 1 Introduction

Olive oil (OO) is a good source of bioactive compounds which are associated with a range of health benefits. More than 30 different phenolic compounds have been identified in OO. These bioactive “phytochemicals” or “phytonutrients” range from 1% to 2% of the total weight content of OO (Covas et al. 2015), and they include phenolic acids, lignans, and flavonoids (Shahidi and Ambigaipalan 2015). A substantial proportion of the olive‐oil phenolic profile comprises derivatives of water‐soluble secoiridoid glycosides, including oleuropein and ligstroside, originating from olive‐fruit tissues. During OO extraction, endogenous enzymatic activity (e.g., β‐glucosidase), hydrolyses these glycosides in olive fruit to form lipophilic oleuropein aglycone and ligstroside aglycone (Ramírez et al. 2016; Velázquez‐Palmero et al. 2017) which are the main phenolic compounds in extra virgin olive oils (EVOO). Oleuropein aglycone is of interest due to its biological properties. These aglycones can undergo further ester hydrolysis (esterase cleavage) to the non‐phenolic elenolic acid and phenolic alcohols; hydroxytyrosol or tyrosol (Jimenez‐Lopez et al. 2020). It has also been suggested that the formation of tyrosol and hydroxytyrosol may also occur spontaneously (Briante et al. 2000). Oleacein and oleocanthal are also present in EVOO: hydrolysis of the methyl esters in oleuropein and ligstroside aglycones (Starec et al. 2021) generates easily decarboxylated carboxylic derivatives (Diamantakos et al. 2020), which undergo simultaneous opening of the secoiridoid ring, leading to their formation (Nikou et al. 2022).

In 2011, the panel on Nutrition, Novel Foods, Food Allergens (part of the European Food Safety Authority, EFSA) concluded that there is evidence for a cause‐and‐effect relationship between the consumption of bioactive phenolic compounds in OO and the protection of low‐density lipoprotein (i.e., cholesterol particles) from oxidative damage in human blood (Papastavropoulou et al. 2022). The specific compounds identified by EFSA are hydroxytyrosol, tyrosol as well as more complex high‐molecular mass polyphenols known as secoiridoids from which hydroxytyrosol and tyrosol can originate (EFSA Panel on Dietetic Products, Nutrition and Allergies (NDA) 2011; López‐Huertas et al. 2021). The EFSA now allows acknowledgment of health claims based on OO phenolic content due to these findings. The EU Regulation (European Commission 2012) details the list of health claims, and the conditions and restrictions of their use. This regulation permits health claims for benefits of the daily ingestion of OO such as EVOO that are rich in phenolic compounds. To claim that OO polyphenols contribute to the protection of blood lipids from oxidative stress, the OO must contain at least 5 mg of hydroxytyrosol and its derivatives (e.g., oleuropein complexes and tyrosol) per 20 g of olive oil (i.e., 250 mg/kg or 0.025%) (Bongiorno et al. 2023; European Commission 2012). The difficulty lies in the fact that the concentration of phenolic compounds can vary between batches of OO and may be below the threshold to obtain health benefit certainty. Enrichment of these compounds in OO via liquid–liquid extraction (Japón‐Luján and Luque de Castro 2008), solid–liquid extraction (Japón‐Luján et al. 2008), or a combination of these procedures is commonly used to meet the threshold; often waste products within the chain, such as olive leaves are used for this purpose. However, the variables within these processes remain understudied; notably, almond flour/meal has not previously been considered as a crude, accessible source of β‐glucosidase for the hydrolysis of oleuropein for enrichment purposes. This study was therefore designed to address these gaps and explore how such an enzymatic conversion might be controlled to support the production of secoiridoid‐enriched OO.

Olive leaf waste is generated during the processes of pruning olive trees, collection of olive fruits, and cleaning‐blending steps in extraction of OO (Topuz and Bayram 2022). About 25 kg of twigs and leaves per tree are accumulated annually by pruning olive trees, and a large amount (about 10% of fruit weight) is separated from the fruits during oil extraction (Selim et al. 2022). Olive leaves contain considerable amounts of phenolic compounds, for example, simple phenols (phenylethanoids, hydroxybenzoic, and hydroxycinnamic acids) and flavonoids (flavones, flavanones, flavonols, and 3‐flavanols), and have higher levels of oleuropein than any other part of the olive tree (Sahin and Bilgin 2018). Oleuropein is reported to reach concentrations of 60–90 mg/g in dried leaves (Omar 2010). This presents an opportunity to utilize the leaves as a source of oleuropein to enhance the bioactive component of OO to a desired concentration.

Oleuropein is responsible for the bitter taste of leaves, which together with the products of its enzymatic hydrolysis (e.g., oleuropein aglycone) have the greatest biological interest (Souilem et al. 2017). Hydrolysis of oleuropein can occur either via the endogenous source of β‐glucosidase or can be more carefully controlled using an exogenous source of β‐glucosidase. The most common source of this enzyme is from sweet and bitter almonds ( *Prunus amygdalus* ) (Guggenheim et al. 2018). With large amounts of olive leaves going to waste each year this is an underutilized source of oleuropein which can be extracted and hydrolysed to desired secoiridoid derivatives such as oleuropein aglycone and subsequently infused into OO. By steeping olive leaves in water, it is possible to produce an olive leaf aqueous extract (OLAE), from which oleuropein can be enzymatically converted by β‐glucosidase to the more lipophilic oil soluble oleuropein aglycone. However, the chemistry of transferral of the aglycone from water is complex and has not been well studied. It is important to note that according to the International Olive Council definition, virgin olive oil is obtained solely from olives by mechanical or physical means and may not undergo treatments other than washing, decantation, centrifugation and filtration (International Olive Council, n.d.). Consequently, the addition of exogenous phenolic extracts could compromise classification of the product as virgin olive oil. However, enrichment of oil is not prohibited and oil with exogenous phenolic compounds could be marketed under a different product designation. In this study, we have endeavored to (1) investigate the extraction of oleuropein from the leaves into an olive leaf aqueous extract and determine its stability; (2) determine if various sources of almond are a suitable source of β‐glucosidase for hydrolysis of oleuropein and (3) assess the transfer of oleuropein aglycone from OLAE into oil. This work is an important step in filling gaps in the literature to learn more about the possibility of producing olive oils that are enriched in secoiridoids and other valuable bioactive compounds at concentrations beneficial for human health.

## 2 Materials and Methods

### 2.1 Olive Leaves

Olive leaves were collected from properties in Waitao, Tauranga, and Claudelands, Hamilton, New Zealand when required during late summer/early Autumn. The tree species were not taxonomically identified. Leaves were either used fresh or left to dry in an incubator at room temperature (RT, 24°C) or oven (70°C) to constant weight, then placed into a brown paper bag and stored in a dry, dark cupboard until use.

### 2.2 Solvents, Standards, Reagents, and Consumables

#### 2.2.1 Solvents

Type 1 water (distilled and deionized; 18.2 MΩcm resistivity), and distilled water were made in‐house at the University of Waikato. Methanol (MeOH, Optima LC–MS grade > 99.99%), acetonitrile (ACN, HPLC grade ≥ 99.99%), and formic acid (Optima LC–MS grade ≥ 99.0%) were obtained from Fisher Chemical. Acetic acid (glacial, *ReagentPlus*, ≥ 99.0%) was obtained from Sigma Aldrich. Acetone (HPLC grade ≥ 99.5%) was obtained from J.T Baker.

#### 2.2.2 Standards and Reagents

Oleuropein (≥ 80.0%) was obtained from Sigma Chemicals and tyrosol (98.5%) was obtained from Aldrich Chemicals. Elenolic acid (> 90.0%), oleacein (90.0%), oleocanthal (95.0%), hydroxytyrosol (98.0%), and oleuropein aglycone (95.0%) were from Toronto Research Chemicals (Canada). Ligstroside (97.0%) was obtained from Biosynth. d‐(+)‐glucose (≥ 99.5%) was obtained from Sigma Aldrich. Sodium chloride (NaCl) was obtained from LabServ (Thermo Fisher Scientific). Almond flour (*n* = 2) and whole raw almonds were obtained from local supermarkets in New Zealand.

### 2.3 Oil Preparation for Analysis

The oil sample preparation was a modification of a published method (Pizarro et al. 2013). In short, 2 mL of MeOH/water (80:20 *v*/*v*) was added to 0.100 g of OO in an Eppendorf tube, vortexed for 1 min, then centrifuged for 5 min at 11,300 RCF. This procedure was repeated 3 times in total; each time, 1.8 mL of supernatant was taken and added to a 15 mL centrifuge tube. The 15 mL tube was then vortexed for 30 s, and a 1 mL sample was taken for analysis in an amber HPLC vial. If samples could not be analyzed within the same day, they were left in a chiller (4°C) and analyzed within 48 h.

### 2.4 Preparation of Olive Leaf Aqueous Extract

To prepare leaves for use, leaves were hand picked off the olive branches; torn leaves were not used due to potential release of endogenous enzymes (Charoenprasert and Mitchell 2012; Johnson et al. 2018). To investigate how leaf drying temperature affected the concentration of oleuropein in OLAE, drying experiments were undertaken using fresh olive leaves (without drying) and leaves dried at 24°C in an incubator or 70°C in an oven until constant weight.

The OLAE was made using leaves and distilled water in a 1:10 ratio. Olive leaves (50–100 g) were placed in a nylon mesh (100 μm) filter sock, suspended in a beaker on a hot plate. Boiling water was poured onto the leaves. The leaves were mixed with a glass mixing rod to ensure all material was in contact with the water; tin foil was placed over top of the beaker to prevent heat and moisture loss. The water was brought back to boiling (100°C ± 5°C) and the leaves were steeped (30 min) while maintaining a temperature of 100°C, with mixing every 5 min. The extract was cooled for 10 min. The filter sock was then removed and the extract was allowed to drain into the beaker for 15 min. Remaining liquid in the filter sock was expressed and the OLAE thus prepared was stored at −20°C until use.

### 2.5 Almond Meal/Milk

To produce almond meal, whole raw almonds (10–20) were blended to a fine powder using a Nutribullet 2.01200 W (2 min). The resulting meal was stored in an airtight tube until use. Almond milk was prepared by adding almond meal with distilled water (1:5 ratio); the mixture was gently shaken on the orbital shaker (15 min). The resulting liquid was filtered with a Büchner funnel with a Schleicher and Schuell filter paper 604 (55 mm). The resulting almond milk was stored in the freezer (−20°C) until use and was kept no longer than 3 months.

### 2.6 Triple Quadrupole Liquid Chromatography Tandem Mass Spectrometry

The Triple Quadrupole Liquid Chromatography Tandem Mass Spectrometry (LC‐QqQ‐MS/MS) system used for both oil and OLAE analysis was an ExionLC high‐performance liquid chromatography (HPLC)/ultra‐high‐performance liquid chromatography (UHPLC) system with an ExionLC AD Multiplate Autosampler and a SCIEX Triple Quad 6500 tandem mass spectrometer. The instrumentation was controlled by Analyst software (Version 1.7.3). MultiQuant software (version 3.0.3) was used for the quantitative and qualitative workflow analysis.

Compounds were separated using a Poroshell 120 EC‐C_18_ column (2.7 μm, 2.1 × 50 mm, Agilent Technologies, USA) fitted with a Phenomenex KrudKatcher Ultra. The column compartment and autosampler were kept at 50°C and 15°C, respectively. The mobile phase consisted of a gradient program using 0.1% acetic acid in Type 1 water (A) and 100% MeOH (B) as follows: 0 min, 10% B; 0.5 min, 10% B; 3 min, 95% B; 4 min, 95% B; 4.10 min, 10% B; with a final time of 5 min and a flow rate of 0.6 mL/min. The injection volume was 2 μL and the needle wash solution used between injections was MeOH/Type 1 water (50:50, *v*/*v*).

The retention times for the target analytes are summarised in Table 1. Oleuropein aglycone had several unresolved peaks corresponding to isomers, and quantification was carried out using the sum of the four peaks. Elenolic acid was a broad peak whose shape often varied with sample type. Therefore, quantification was carried out using the sum of elenolic acid peaks between 1.6 and 3.5 min (regardless of peak shape) to be consistent when reporting results. Method validation data for olive oil and olive leaf aqueous extract are presented in Data S1–S5.

**TABLE 1:** Summary of MS parameters for the determination of OO and OLAE target analytes.

Analyte	Retention time (min)	Precursor ion (*m*/*z*)	Product ion (*m*/*z*)	DP^a^ (V)	EP^b^ (V)	CE^c^ (V)	CXP^d^ (V)
Oleuropein	2.98	539	−307 **−275**	−101 −124	−6	−31 −28	−15 −14
Oleocanthal	3.13	303	**−179** −165	−45	−6	−10 −14	−9 −7
Oleacein	2.79	319	**−195** −183	−60	−12	−16 −10	−16 −10
Hydroxytyrosol	0.63	153	**−123** −122	−60	−14	−19 −28	−14 −12
Tyrosol	1.04	137	**−119** −106	−19	−19	−20 −21	−13 −14
Oleuropein aglycone	3.40, 3.47, 3.60, 3.79	377	**−307** −275	−58	−12	−15 −17	−9 −8
Ligstroside	3.24	523	**−361** −291	−100	−8	−20 −31	−11 −9
Elenolic acid	1.6–3.5	241	**−127** −95	−30	−10	−16 −14	−15 −11

*Note:* Product ion transitions used for quantification are shown in bold text.

^a^ DP: declustering potential (V).

^b^ EP: entrance potential (V).

^c^ CE: collision energy (V).

^d^ CXP: collision cell exit potential (V).

The mass spectrometer (MS) was operated in ESI negative ion mode using multiple reaction monitoring (MRM), with a retention time window of 4.003 min and a target scan time of 0.2502 s. The following conditions were used: IonSpray Voltage, −4500 V; ion source gas 1, 70 psi; ion source gas 2, 60 psi; curtain gas, 25 psi; collision gas, 8 psi; dwell time, 5 milliseconds; and a flow rate and drying gas (nitrogen gas, N_2_) temperature of 10 L/min and 450°C, respectively. The precursor ion, two MRM transitions with best signal‐to‐noise (quantifier and qualifier) and optimised MS parameters are summarised in Table 1.

Due to suppression and enhancement effects on MS/MS, multiple dilutions were carried out for each sample (up to 4, using Type 1 water). The number of dilutions required was dependent on the concentration of target analytes in different OLAE samples analyzed ranging between 20 and 20,000‐fold dilutions. The concentration of analytes in OLAE is reported as the average of the dilutions having analyte concentrations consistent within ±10%. Limits of detection and quantitation can be found in Data S1.

### 2.7 Data Analysis and Graphical Presentation

Analysis of data and graphical visualization were carried out using Microsoft Office Excel (Version 2303), Minitab (Version 21.4.1) and RStudio (Version 2026.01.0). For statistical calculation, samples that had an analyte concentration less than limit of detection (<LOD) were treated as a missing value for summary statistics (e.g., averages). Chemical structures, diagrams and reactions were produced using PerkinElmer ChemDraw (Version 21.00).

## 3 Results and Discussion

### 3.1 Extraction of Oleuropein From Olive Leaves

#### 3.1.1 Effects of Drying Leaves

Olive leaves are an industry waste product during olive oil manufacture and contain a high concentration of oleuropein. To extract the oleuropein, an aqueous extraction was carried out to produce an olive leaf aqueous extract (OLAE) and was used as the medium to convert oleuropein to oleuropein aglycone before transferring the aglycone to the oil. To determine if processing the leaves influenced the concentration of compounds extracted, both intact fresh and dried leaves were used to create an OLAE that was high in oleuropein. Intact leaves were used to preserve the oleuropein in its glycoside form, as any injury to olive leaf tissues—whether by pathogens, herbivores or mechanical damage—disrupts cellular structure, allowing endogenous β‐glucosidase to hydrolyse oleuropein and ligstroside glycosides to their aglycone forms (Charoenprasert and Mitchell 2012; Hachicha Hbaieb et al. 2015; Johnson et al. 2018). Initially, a gentle drying method was employed (24°C until constant weight) and the extraction efficiency was compared to fresh leaves (Table 2). The hydrophobic oleuropein aglycone was measured in OLAE created from leaves dried at 24°C, albeit at a low concentration (< 22 mg/kg). The moisture content of the leaves ranged between 52% and 56% which albeit slightly higher, was similar to other studies which ranged between 46% and 50% (Boudhrioua et al. 2009; Cabrera‐Gómez et al. 1992; Ramírez et al. 2023). A higher concentration of oleuropein glycoside was observed when OLAE was made from dried leaves compared to fresh leaves. Of all compounds, it had the highest absolute increase (421 mg/kg) which accounted for a 23% increase since the concentration of oleuropein is several orders of magnitude higher than other compounds analyzed. A higher concentration of hydroxytyrosol was also extracted when using dried leaves (134%, 388 mg/kg). The increase in compounds corroborates other investigations of phenolic compounds in olive leaf extracts (Boudhrioua et al. 2009; Ghomari et al. 2019; Kamran et al. 2015). Higher concentrations of phenolic compounds in the OLAE made from dried leaves are likely due to the cell walls bursting, facilitating the liberation of the phenolic compounds. Similar trends have also been observed in other leaf varieties (e.g., tomatoes (Chang et al. 2006) herbal materials (Kozłowska et al. 2021) and stinging nettle (Garcìa et al. 2021)). The results suggest that a leaf drying process tends to enhance the functional value of resulting products.

**TABLE 2:** Extraction of compounds into olive leaf aqueous extract when prepared using leaves dried at 24°C (batch 1) and fresh leaves. Results are presented as the average of dilutions undertaken ± SD (*n* = 3).

Analyte	Method of drying			Percentage difference (%)
	24°C, 14 days (dry weight, mg/kg)	24°C, 14 days (wet weight equivalent, mg/kg)	Fresh (wet weight, mg/kg)	Dried vs. fresh leaves
Oleuropein	3667.5 ± 28.7	2055.9 ± 16.1	1634.9	23
Oleuropein aglycone	17.3 ± 0.14	<LOD	4.7 ± 0.2	69
Oleacein	3.5 ± 0.3	3.7 ± 0.1	<LOD	—^a^
Oleocanthal	<LOD	<LOD	<LOD	—^a^
Ligstroside	663.0 ± 7.9	371.7 ± 4.4	257.3 ± 16.3	36
Hydroxytyrosol	841.7 ± 11.4	483.8 ± 12.2	95.8 ± 1.7	134
Tyrosol	30.6 ± 2.6	17.2 ± 1.5	1.4 ± 0.04	170
Elenolic acid	122.7 ± 3.6	68.8 ± 2.0	23.7 ± 3.0	97

^a^ —: a % difference could not be calculated due to one value being <LOD.

To determine if drying leaves at an elevated temperature influenced the concentration of phenolic compounds in the OLAE, a second batch of leaves (with different concentrations of compounds compared to batch 1) was dried at 24°C and 70°C (Table 3). Higher concentrations of oleuropein and ligstroside were observed when leaves were dried at 70°C; whereas concentrations of elenolic acid, oleuropein aglycone, tyrosol, oleocanthal and oleacein were lower. This pattern is consistent with the proposed degradation pathway in which oleuropein and ligstroside are hydrolysed to their respective aglycones and subsequently broken down into smaller phenolic compounds. Similar trends have been reported in the literature. For example, Şahin et al. reported higher oleuropein concentrations in leaves dried at 100°C for 45 min compared with lower temperature with longer drying times (50°C for 270 min and 80°C for 75 min). Increased oleuropein concentrations at elevated drying temperatures have also been observed in other studies employing a range of drying conditions, including 60°C for 48 h and 105°C for 3 h (Kamran et al. 2015; Malik 2008). These observations have been attributed to thermal denaturation of endogenous enzymes, particularly β‐glucosidase, which reduces enzymatic degradation of oleuropein during processing.

**TABLE 3:** Extraction of compounds into olive leaf aqueous extract when prepared using leaves (batch 2) dried at 24°C and 70°C. Results are presented as the average of dilutions undertaken ± SD (*n* = 3).

Analyte	Drying temperature		Percentage difference (%)
	70°C, 3 days (dry weight, mg/kg)	24°C, 14 days (dry weight, mg/kg)	70°C vs. 24°C
Oleuropein	1744 ± 7.7	1167 ± 10.3	40
Ligstroside	906 ± 4.8	325 ± 2.6	94
Oleuropein aglycone	4.9 ± 0.1	22.1 ± 0.9	127
Oleacein	<LOD	76.2 ± 0.5	—^a^
Oleocanthal	<LOD	6.3	—^a^
Hydroxytyrosol	781 ± 18.0	1890 ± 10.6	83
Tyrosol	10.7 ± 0.2	36.3	109
Elenolic acid	198 ± 9.6	268.8 ± 0.5	31

^a^ —: a % difference could not be calculated due to one value being <LOD.

Despite this general trend, conflicting results have also been reported, with some studies observing greater oleuropein concentrations following drying at lower temperatures. One explanation is the differences in sample preparation. In Al‐Rimawi et al. (2015), leaves were ground prior to extraction, a process that may have activated the endogenous β‐glucosidase and also increased surface area, promoting the hydrolysis of oleuropein to oleuropein aglycone and the subsequent formation of hydroxytyrosol and elenolic acid (Figure 1). However, similar trends have also been observed in studies that did not grind the leaves before extraction (Al‐Rimawi et al. 2015; Cör Andrejč et al. 2022; Hata 2004), suggesting a combination of factors influences the final concentration of compound within the pathway.

**FIGURE 1:**
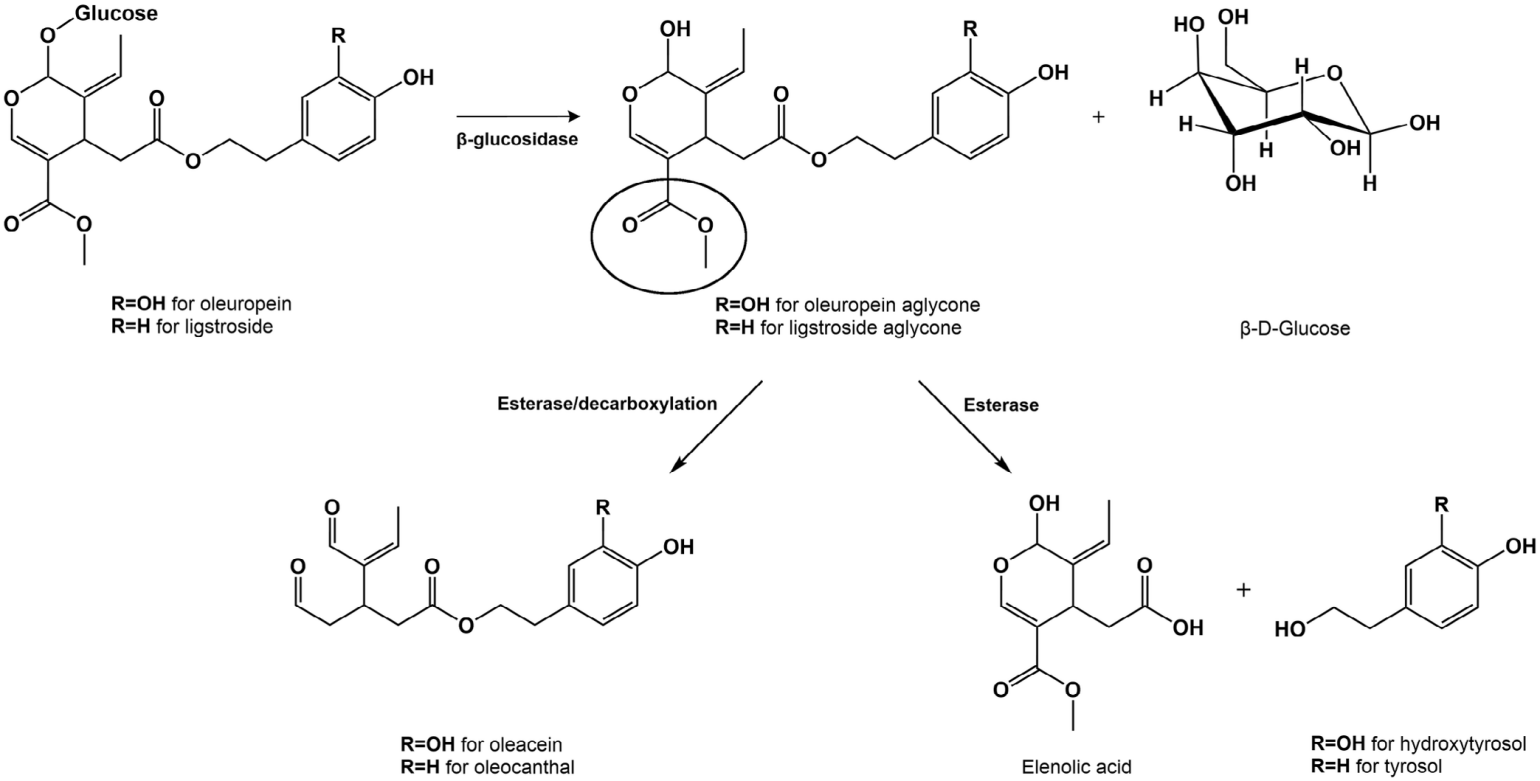
The pathway from oleuropein and ligstroside to their respective aglycones, and then to their secoiridoid derivatives by endogenous enzymatic hydrolysis and decarboxylation. The methyl ester has been circled on the aglycone to show which group is hydrolysed prior to decarboxylation to produce oleacein and oleocanthal.

Drying temperature and drying duration appear to be particularly important variables. For example, Hata (2004) reported higher oleuropein concentrations in leaves dried at temperatures above 80°C than in leaves dried at lower temperatures, further supporting the idea that elevated temperatures reduce β‐glucosidase activity through thermal degradation.

Consideration of time, temperature and leaf integrity helps explain the results in the current study; whole leaves dried at 70°C reached complete dryness after 3 days, while leaves dried at 24°C required 14 days. The longer drying duration may have provided greater opportunity for endogenous β‐glucosidase to convert oleuropein and ligstroside to their respective aglycones, resulting in lower concentrations of the parent compounds. These results highlight the importance of regulating the preparation method to achieve consistent extraction of compounds and to regulate further pathways.

In both batches of OLAE made with leaves dried at 24°C, hydroxytyrosol, tyrosol and elenolic acid were higher compared to OLAE made with fresh and 70°C dried leaves. Native hydroxytyrosol, tyrosol and elenolic acid rarely exist in their free form, and their formation requires enzymatic ester hydrolysis. Hydrolysis of oleuropein aglycone forms hydroxytyrosol, and hydrolysis of ligstroside aglycone forms tyrosol, while elenolic acid is a hydrolysis product of both analytes (De Leonardis et al. 2008). The length of drying at 24°C (> 5 days) allowed time for the formed aglycones to be further enzymatically hydrolysed into their respective derivatives hydroxytyrosol, tyrosol and elenolic acid. This demonstrates that drying time and temperature can be exploited to produce an OLAE with the desired compound profiles. Across the two leaf batches examined, oleuropein concentrations in OLAE followed the general order fresh < room‐temperature dried < 70°C dried.

#### 3.1.2 Stability of Analytes in Olive Leaf Aqueous Extract (OLAE)

The analytes in OLAE were examined for stability. Olive leaves were boiled for 30 min per the method described in Section 2.4 to inactivate the endogenous enzymes to prevent hydrolysis of the glycosides. Samples were stored at −20°C, 24°C, and 40°C and periodically analyzed over time (up to 189 days, Figure 2). Results for all analytes at each time period are presented in Data S6–S8. All compounds remained stable when stored at −20°C.

**FIGURE 2:**
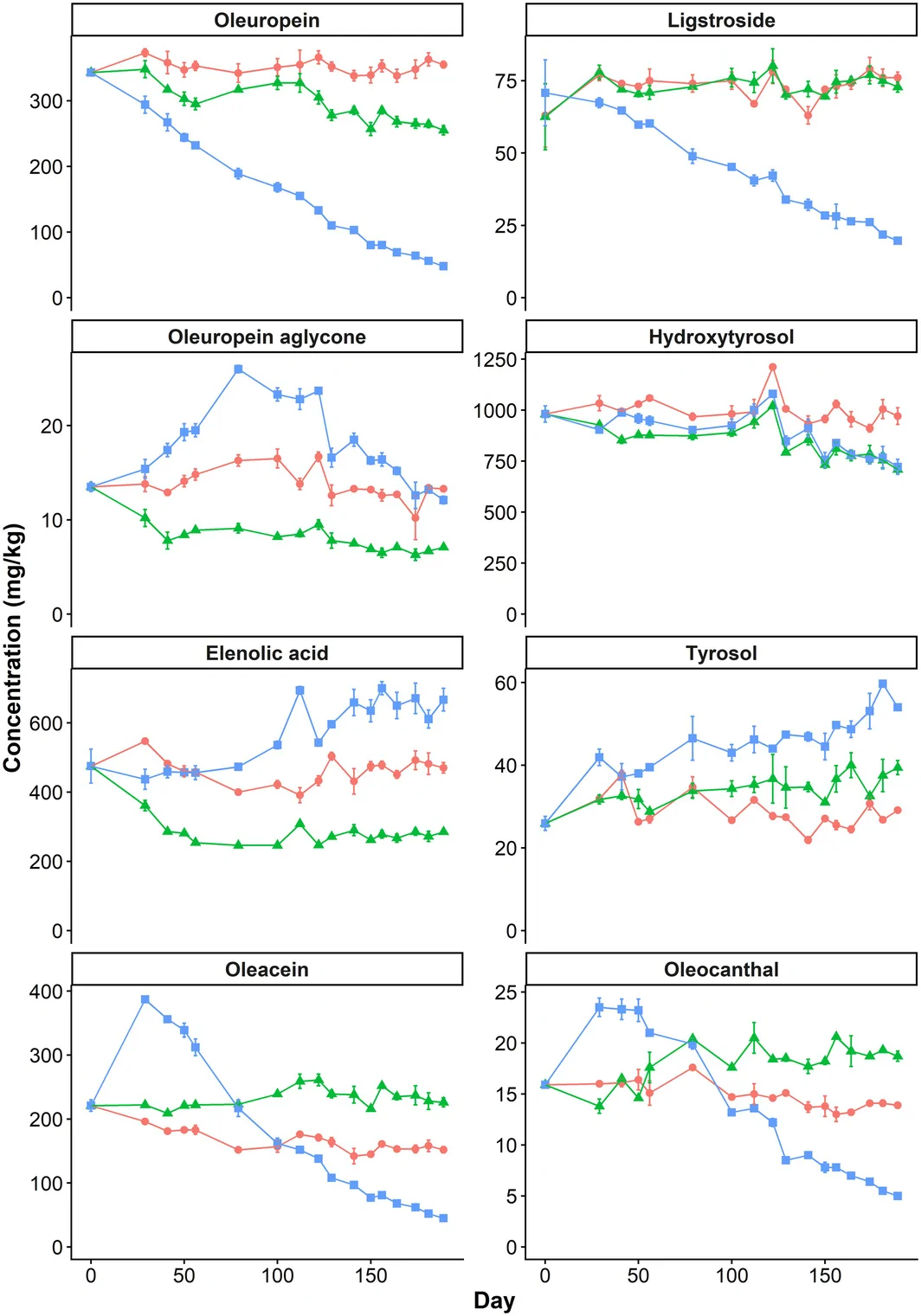
Concentration of compounds (mg/kg) versus time for olive leaf aqueous extracts stored at −20°C (red circle), 24°C (RT, green triangle), and 40°C (blue square) for 189 days. The error bars are set to the SD of duplicate samples thus results are presented as the average ± SD (*n* = 3).

At 24°C, the initial concentration of oleuropein was 343 ± 6 mg/kg and decreased by 26% over 189 days, whereas at 40°C, it decreased by 29% after 50 days and by 84% after 189 days. Although the zero‐order model showed a slightly higher coefficient of determination (*R* ^2^ = 0.9878) than the first‐order model (*R* ^2^ = 0.9718), both models provided a good description of the experimental data. The similarity in goodness‐of‐fit suggests that the available data do not allow a straightforward discrimination between zero‐ and first‐order kinetics on the basis of *R* ^2^ alone. Therefore, the assignment of first‐order kinetics was made primarily on the basis of consistency with previous studies (Gonzalez‐Ortega et al. 2023; Martínez‐Navarro et al. 2021; Stamatopoulos et al. 2014). Oleuropein and ligstroside can be hydrolysed by β‐glucosidase to their respective hydrophobic aglycones which can undergo further hydrolysis (Figure 1) giving products with intermediate solubility. However, following the thermal inactivation of endogenous enzymes, oleuropein likely underwent degradation at 40°C via mechanisms such as chemical oxidation, cleavage of covalent bonds or active groups undergoing side reactions such as ring closure and polymerization (Attya et al. 2010). These results contrast with previous literature, where greater reductions in oleuropein were observed, which may be due to the different preparation techniques of leaves before storage (Martínez‐Navarro et al. 2021).

Unexpectedly, a low concentration of the hydrophobic oleuropein aglycone was detected in the aqueous OLAE (13.5 ± 0.5 mg/kg at the start of storage). At 24°C, the aglycone decreased by 50% over 189 days, likely due to oleuropein aglycone degrading to oleacein, elenolic acid and hydroxytyrosol, all of which increased over time. In contrast, at 40°C, oleuropein aglycone initially increased by 86% after 79 days (presumably due to the thermal degradation of oleuropein); after which, the aglycone concentration declined, reaching a 50% reduction after 189 days, likely due to degradation to oleacein and elenolic acid, which increased over time. At 40°C, hydroxytyrosol did not show the expected increase and may have undergone degradation or oxidation; this has also been previously observed in an olive fruit brine solution (Ramírez et al. 2016). Adsorption of oleuropein aglycone to the storage vessel may also have contributed to the observed decrease, given its hydrophobic character.

In contrast to oleuropein, ligstroside was stable at 24°C but decreased when stored at 40°C (68% after 189 days, 63 ± 11 mg/kg starting concentration). Ligstroside has a similar structure to oleuropein but the lack of an adjacent hydroxyl in ligstroside prevents its simple conversion to an *o*‐quinone which may account for its increased stability at the lower storage temperature and slower decrease at 40°C (68% vs. 84% after 189 days). *Ortho*‐diphenols, such as oleuropein, are more reactive and can be easily converted to *ortho*‐quinones through a radical reaction (Hvattum and Ekeberg 2003). Ligstroside also showed strong linearity of a zero‐order plot (*R* ^2^ = 0.9857), and reasonable linearity for a first‐order plot (*R* ^2^ = 0.9640).

At 24°C, elenolic acid decreased until Day 79, after which time the concentration plateaued. The change in elenolic acid concentration was temperature dependent due to its formation from oleuropein aglycone and/or ligstroside aglycone. A higher concentration of elenolic acid was present at 40°C (29% increase after 189 days) compared to 24°C, where it decreased. Hydroxytyrosol, a derivative of oleuropein aglycone ester hydrolysis, did not increase over time.

Results of the stability trial of OLAE at different temperatures (−20, RT, 40°C) for 189 days demonstrated that most compounds remained stable at −20°C. At room temperature, compounds either tended to decline (oleuropein) or remained stable (ligstroside) over 189 days, whereas precursors such as oleuropein and ligstroside degraded significantly at 40°C, consequently resulting in increased concentrations of their respective derivatives. These findings emphasize the importance of low‐temperature storage to preserve phenolic integrity and minimize the formation of degradation products. This ensures OLAE retains a higher concentration of oleuropein for controlled enzymatic conversion to its aglycone. This will assist producers in optimizing the temperature and duration of the hydrolysis process to maximize the conversion of glycosides into lipophilic aglycones for fortification of OO.

### 3.2 Almond as a Source of β‐Glucosidase

Olive leaves contain an endogenous source of β‐glucosidase; however, to ensure sufficient enzyme activity is present for rapid hydrolysis, exogenously supplied enzyme can be used to accelerate the reaction of oleuropein and ligstroside to their respective aglycones. This may be a useful strategy from the perspective of achieving ultimate product‐to‐substrate ratios and scaling the process up. β‐glucosidase that originates from almonds has been the subject of many kinetic studies relative to other glucosidases; it is easily isolated in large quantities from a readily available material and is commercially available. However, β‐glucosidase extracted and purified from almonds or bitter almonds is expensive, and the purification methods that employ buffers such as ammonium sulfate and ammonium hydroxide would render the final product unsuitable for human consumption (Jin et al. 2019). Therefore, this study investigated edible sweet almond flour and almond meal as crude, food‐compatible sources of β‐glucosidase for oleuropein hydrolysis—an approach that to the authors' knowledge, has not previously been reported.

A range of optimum working temperatures up to 60°C have been reported for β‐glucosidase activity (Hati et al. 2020; Montero and Romeu 1993; Olajuyigbe et al. 2016; Sigma 1994). 40°C was selected in this research because it was the most commonly reported temperature (Hati et al. 2020; Montero and Romeu 1993; Sigma 1994). At the end of each experiment, the enzyme was inactivated by the addition of methanol (1:1, v/v), which successfully halted any further conversion of compounds before analysis.

To assess whether almond flour could effectively convert oleuropein, 100 mg was added to OLAE (1 mL) containing 3668 mg/kg oleuropein. Near complete conversion of oleuropein and ligstroside (> 99% reduction) was achieved after 12 h (Table 4). While minimizing the quantity of almond flour used is commercially advantageous in terms of cost reduction, optimisation of the flour to substrate ratio was beyond the scope of this study and the quantity used was sufficient to demonstrate near‐complete conversion of oleuropein.

**TABLE 4:** Change in oleuropein and ligstroside in OLAE (1 mL) with addition of almond flour (100 mg) as source of β‐glucosidase at 40°C.

	0 min	0.5 h	12 h
	mg/kg		
**Oleuropein**	3668 ± 30	1954 ± 4	40.9 ± 0.3
**Ligstroside**	585 ± 30	270 ± 20	<LOD
Almond flour added (mg)	—	100	100

The effectiveness of β‐glucosidase from different batches of almond flour (made from either blanched or skinned almonds) and laboratory‐made almond meal (produced from raw almonds without blanching) was investigated to determine differences in enzymatic activity. Differences were observed in the amount of oleuropein and ligstroside converted when using different batches of almond meal/flour; for oleuropein, decreases ranging from 648 to 2904 mg/kg were observed in 30 min. Under the conditions tested, freshly prepared almond meal produced a 4.5‐fold greater reduction in oleuropein and ligstroside concentrations after 30 min than the oldest almond‐flour (almond flour 2). The differences in activity between the powders are likely attributable to the difference in processing methods; to produce both almond flour and almond meal, freshly picked raw almonds are steam pasteurized before being passed through a series of dryers to cool them and reduce the moisture content (Bennett 2016). An additional step is carried out to produce almond flour—the almonds are blanched (> 100°C, 2–5 min) to remove the brown skins (pellicle) before milling into flour (Bennett 2016). The elevated‐temperature blanching treatment may have partially denatured β‐glucosidase, potentially accounting for the lower conversion rates observed with almond flour. These findings highlight the importance of sourcing fresh, unblanched almond meal or alternative enzyme preparations to maximize hydrolytic efficiency and minimize processing time.

Overall, these findings provide a foundation for developing scalable, cost‐effective strategies to facilitate the enzymatic hydrolysis of oleuropein for functional food and nutraceutical applications.

### 3.3 Effect of Temperature on β‐Glucosidase Activity

Preliminary work suggested that the small amount of oleuropein aglycone formed in the OLAE was likely establishing a favorable interaction with the lipids present on the surface of the almond flour/meal which account for ~50% of almonds' composition (Barreca et al. 2020), thus hindering the potential mass transfer into the oil in subsequent experiments. As almond milk is filtered and therefore contains less particulate matter with which the aglycone could interact, it was used as the source of β‐glucosidase to investigate the effect of temperature (30°C, 40°C, 50°C, and 60°C) on the hydrolysis of oleuropein and to determine whether the hydrolysis conditions could be optimized within the OLAE.

Results from the temperature experiments showed that oleuropein and ligstroside decreased faster with increasing temperature. After 120 min at 30°C, oleuropein and ligstroside decreased by 1499 and 331 mg/kg respectively, compared to 3175 and 561 mg/kg respectively at 60°C. This contrasts with a previous study reporting that soy derived β‐glucosidase is inactivated at 60°C under comparable pH used in the current study (Matsuura and Obata 1993).

As previously mentioned, the hydrophobic oleuropein aglycone was detected in the aqueous phase and at each temperature, the aglycone increased to a maximum in the OLAE before decreasing. Additionally, temperature affected the amount of aglycone that was produced; less compound was formed at a higher temperature (at 10 min a maximum of 613 mg/kg at 40°C compared to 357 mg/kg at 60°C). The lower aglycone concentrations observed at 50°C and 60°C may reflect increased degradation of oleuropein aglycone as it formed, as aglycones are generally less stable than their glycoside precursors (Johnson et al. 2018). Possible explanations for the loss of the aglycone in the OLAE after 10 min include poor stability and solubility in the aqueous phase, adhesion to the vessel or its conversion to other products in the reaction pathway. One complication with hydrophobic compounds is their low solubility and stability in aqueous media. Furthermore, hydrophobic compounds can easily adsorb onto commonly used plastic and glassware altering the solution concentration measured (Dincel et al. 2023); this phenomenon may account for the observed decline at both temperatures after 10 min. However, it is more probable that the higher temperature converted the aglycone to an *ortho‐*quinone via a radical reaction, due to the reactive *ortho‐*diphenol group (Johnson et al. 2018), thus lowering the overall concentration of aglycone that could transfer to the oil. A similar trend was observed when olive oil was sautéed domestically, with a larger decrease in secoiridoids when a higher temperature was used (Lozano‐Castellón et al. 2020). Accordingly, an optimal temperature must balance the rate of glycoside hydrolysis with the stability of the resulting aglycone product.

Hydroxytyrosol and tyrosol both increased at all temperatures in the OLAE, while elenolic acid increased to a maximum then remained stable over time (Figure 3). The increase was expected for hydroxytyrosol and elenolic acid as the precursor (oleuropein aglycone) for both compounds decreased at all temperatures. The precursor for tyrosol, ligstroside aglycone, was not quantified in this study but is presumed to undergo degradation similar to oleuropein aglycone, contributing to the observed increase in tyrosol.

**FIGURE 3:**
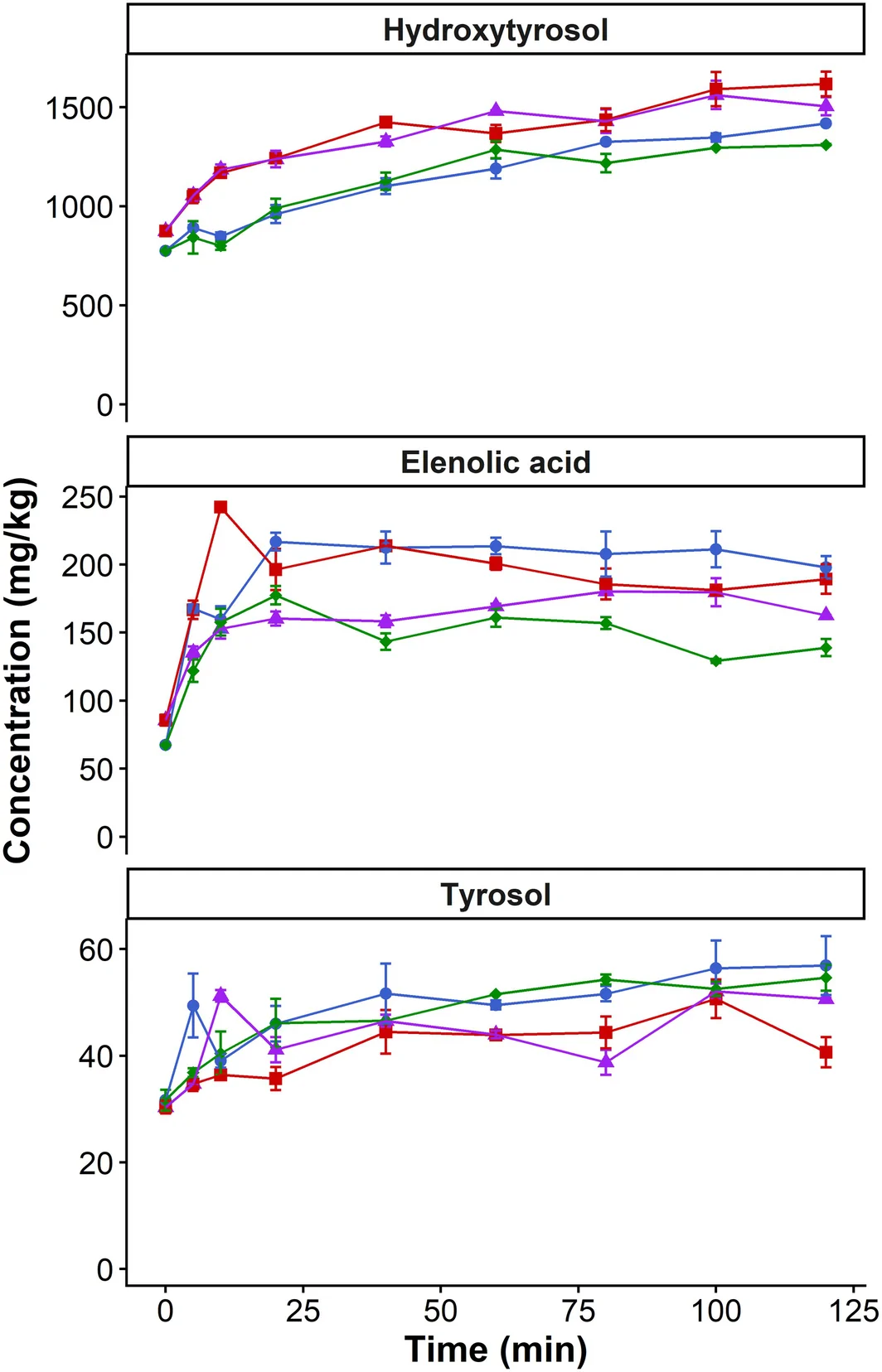
Concentration of (A) hydroxytyrosol, (B) tyrosol, and (C) elenolic acid versus time at 30°C (circle), 40°C (square), 50°C (diamond), and 60°C (triangle) using almond milk as the enzymatic source. Results are presented as the average of duplicate samples (*n* = 2).

To investigate the stability of oleuropein aglycone at elevated temperatures, samples were first hydrolysed at 40°C for 10 min to achieve the maximum oleuropein aglycone concentration, after which β‐glucosidase was inactivated by the addition of water/methanol (1:1, v/v) at each time point. The OLAE was sub‐sampled periodically up until 40 min and analyzed for aglycone content. Heating at 60°C for 40 min decreased the aglycone by 455 mg/kg compared to only 151 and 84 mg/kg at 40°C and 30°C, respectively. Degradation of oleuropein aglycone followed first order kinetics. Kinetic rates and the half‐lives at each temperature are summarized in Table 5. The large difference in half‐life at 30°C and 60°C suggests temperature plays an important role in the stability of the aglycone. To the authors' knowledge, this is the first study to determine the half‐life of oleuropein aglycone in methanol. A previous study reported that oleuropein from leaf extracts was more stable in methanol than water (Malik 2008), suggesting that the half‐life of the aglycone in a purely aqueous extract may be shorter than reported here. In comparison, the aglycone is much more stable in an oil matrix with a reported half‐life of 405 days at 25°C (Lozano‐Sánchez et al. 2013). Overall, these results demonstrate the importance of extracting and rapidly transferring the aglycone from the leaf matrix and transferring it to the oil to minimize degradation losses.

**TABLE 5:** Initial and final concentration (mg/kg), rate constant (k), and calculated half‐life period (t_1/2_) of oleuropein aglycone in 50:50 OLAE:MeOH at 30°C, 40°C, and 60°C. Concentration results are presented as the average of duplicate samples ± SD (*n* = 2).

Temperature (°C)	Oleuropein aglycone concentration (*n* = 2, mg/kg)		Kinetic parameters for oleuropein aglycone	
	Initial (0 min)	Final^a^	k (minutes^−1^)	T_1/2_ (minutes)
30	451 ± 7	200 ± 6	0.003	256
40	353 ± 5	67.1 ± 0.2	0.006	125
60	550 ± 7	95 ± 1	0.044	16

^a^ Experiments at 40°C and 30°C were conducted for 300 min and the experiment at 60°C was conducted for 40 min to achieve at least 85% conversion.

### 3.4 Transfer of Oleuropein Aglycone From Olive Leaf Aqueous Extract to Oil

To enrich the olive oil, the aglycone must be transferred from the aqueous OLAE to the oil. Stepwise hydrolysis of oleuropein in the extract before introduction into the oil was deemed an unsuitable pathway since the aglycone is not stable in aqueous solution. Instead, in a preliminary enrichment experiment, OLAE, almond flour and oil were combined in a vessel for 30 min at 40°C with mixing every 5 min for 30 s. The oleuropein (341 mg/kg) in the OLAE was successfully hydrolysed (94%; 322 mg/kg decrease). However, only 0.3 mg/kg oleuropein aglycone was measured in the oil phase, while 9 mg/kg was measured in the OLAE at the end of the experiment. These phase concentrations indicate substantial apparent loss relative to the decrease in oleuropein concentration. It is possible that the oleuropein aglycone was further enzymatically hydrolysed or degraded into its respective derivatives such as oleacein, hydroxytyrosol and elenolic acid given that the experiment was carried out for 30 min, whereas the maximum amount of oleuropein aglycone was achieved at 10 min (Section 3.3). However, no increase in derivative compounds were measured in the OLAE or oil at the conclusion of the experiment. To improve detection of potential aglycone transfer in subsequent experiments, an OLAE with a higher initial oleuropein concentration was selected (3668 mg/kg). Additionally, as the oleuropein aglycone appeared to be establishing a favorable interaction with the lipids present on the surface of the almond flour, almond flour was substituted for almond milk to remove presence of particulate matter. The period of incubation was also reduced to 10 min as this corresponded to the highest concentration of oleuropein aglycone before degradation (Section 3.3). Despite these modifications the aglycone concentration only reached 6.5 mg/kg in the oil which is well below reported concentrations in oil, which can reach 1786 mg/kg oleuropein aglycone (Różańska et al. 2020). Therefore, the system in this study was not near saturation suggesting that additional factors were contributing to the limited transfer of the aglycone to the oil phase. Interestingly, when OLAE contained 3668 mg/kg oleuropein, the highest aglycone concentration in the extract was observed when almond milk was used as the source of enzyme compared to flour (545 mg/kg vs. 235 mg/kg). This suggests that almond milk altered the aqueous phase and may have increased retention of lipophilic compounds, such as oleuropein aglycone, in the aqueous phase. Additionally, a higher starting concentration of oleuropein in the OLAE contributed to an increased aglycone concentration in the oil.

Due to the limited transfer of oleuropein aglycone to the oil phase, additional experimental conditions were investigated to identify factors limiting mass transfer. Vigorous emulsification using a laboratory homogenizer (LabServ) was applied every 5 min for 30 s over a 30 min period. This produced a more stable emulsion than vortex mixing, however, it did not increase the quantity of aglycone transferred to the oil. To further promote a more stable emulsion the interfacial tension of the emulsion can also be reduced. Interfacial tension is the force acting between two different liquids; a higher interfacial tension means both liquids tend to separate into two phases. Thus, decreasing the interfacial tension is essential to create a stable emulsion and better mass transfer (Lee et al. 2023; Misono 2019). Fatty acids are known to affect interfacial tension (Gmach et al. 2019), and phenolic compounds can also act as surfactants to reduce interfacial tension between immiscible liquids (Anestopoulos et al. 2020; Besha et al. 2018; Lee et al. 2023). Therefore, two EVOOs differing in total phenolic content (23 and 118 mg/kg) were evaluated to determine whether phenolic content influenced aglycone transfer. However, no difference in aglycone mass transfer from the aqueous to oil phase was observed. Again, a higher aglycone concentration (187 and 226 mg/kg) was observed in the aqueous phase of these experiments which may be due to micelle formation facilitated by almond milk, stabilizing the aglycone in the aqueous phase and inhibiting mass transfer into the oil. To determine whether poor mass transfer was due to the aglycone adsorbing onto the particulate matter present in the aqueous phase of enrichment experiments, the aqueous phase was filtered after inactivation of the enzyme. As micelles have a typical diameter between 10 and 100 nm (Koopaie 2020) they would pass through the filters. No significant difference in oleuropein aglycone concentration was observed between non‐filtered and 0.45 and 0.2 μm filtered samples (454 ± 7, 474 ± 26 and 459 ± 14 mg/kg, respectively), thus supporting the hypothesis that a stable micelle structure exists within the aqueous phase of samples due to the presence of oleuropein aglycone.

Salting‐out and sugaring‐out approaches were also evaluated as strategies to promote aglycone transfer to the oil phase; however, no improvement was observed under the conditions tested. It should be noted that care is required during these procedures as elevated concentrations of salt or sugar may alter the solubility, binding, stability, and the biological activities of enzymes, possibly leading to deactivation through changes in water activity. The water activity (a_w_) of saturated NaCl and glucose OLAE solutions at 22°C was 0.75305 ± 0.0002 and 0.9127 ± 0.004, respectively, and therefore remained within a range expected to support enzymatic function. Nevertheless, reduced water activity may still have influenced enzyme activity and substrate solubility.

Collectively, these experiments demonstrated that oleuropein hydrolysis was successful; however, the transfer of the resulting aglycone into the oil phase was consistently inefficient. Even with modifications such as increasing the initial oleuropein concentration in OLAE, substituting almond flour with almond milk, and applying vigorous emulsification, oil enrichment remained minimal. As both enzyme sources achieved complete hydrolysis of oleuropein throughout the experiments, other factors were limiting the mass transfer. The elevated concentration of aglycone in the aqueous phase treated with almond milk relative to almond meal is likely attributable to micelle formation, which would inhibit transfer into the oil layer. Binding to solid components within the enzyme sources may have also contributed to aglycone losses, or further degradation of hydrolysis products may have occurred, although the expected products of these reactions were not observed. Furthermore, filtration experiments confirmed that micelles likely persist even after particulate removal, further supporting the hypothesis that micellar stabilization is a primary barrier to oil enrichment under these experimental conditions. Attempts to manipulate interfacial tension by using OO's with different total phenolic content and applying salting and sugaring out techniques did not improve transfer, indicating that alternative strategies beyond conventional emulsification or concentration adjustments are required. Careful control of the matrix composition will be essential to overcome the complex interactions between the aqueous and oil phases and to optimize aglycone transfer into the oil.

## 4 Conclusion

Olive leaves are a promising source of phenolic compounds and oleuropein for potential olive‐oil enrichment. However, under the conditions tested the mass transfer of oleuropein aglycone into oil was minimal. Almond‐derived β‐glucosidase was effective at hydrolysing oleuropein to its aglycone; however, careful temperature optimisation is critical to balance conversion and stability. Despite successful hydrolysis, oleuropein aglycone transfer from aqueous extract to oil was minimal, likely due to factors such as micelle formation, degradation, and binding to solid components in the aqueous phase. These findings highlight the need for strategies that enhance interfacial transfer and prevent aglycone loss.

## Author Contributions

**Jade te Bogt:** investigation, formal analysis, data curation, methodology, writing – original draft, writing – review and editing. **David Speed:** supervision, methodology, formal analysis, resources, writing – review and editing. **Ralf C. Schlothauer:** conceptualization, supervision, project administration, writing – review and editing. **Jed W. Fahey:** funding acquisition, writing – review and editing. **Megan N. C. Grainger:** supervision, formal analysis, writing – original draft, writing – review and editing.

## Funding

This work was supported by the Ministry of Business, Innovation and Employment (MBIE), Callaghan Innovation (for R&D Fellowship Grant), and Brassica Protection Products LLC.

## Disclosure

J.W.F. is a scientific advisor to BPP as well as to a few other small food companies. None of them are involved in olive leaf extract.

## Supporting information


**Data S1:** Limits of detection and quantification for target analytes when analyzed by LC–MS/MS.
**Data S2:** Endogenous, high and low spike sample concentrations (μg/L) and spike recoveries for olive oil (OO) (%). Results are presented as the average ± SD (*n* = 9).
**Data S3:** OO 1 intra‐ and inter‐day %RSDs for endogenous, low and high spike samples. Intra‐day results (*n* = 3) are presented as the range of %RSDs from each validation day. The inter‐day precision is presented as %RSD over 3 days (*n* = 9).
**Data S4:** OO 2 intra‐ and inter‐day %RSDs for endogenous, low spike and higher spike samples. Intra‐day results (*n* = 3) are presented as the range of %RSDs from each validation day. The inter‐day precision is presented as %RSD over 3 days (*n* = 9).
**Data S5:** Spike recoveries, intra‐day and inter‐day precision ranges for the target analytes using 80 μg/L spiked OLAE data. Spike results are presented as the average ± SD (*n* = 9). Intra‐day results are presented as the range of %RSDs from each validation day (*n* = 3). Inter‐day results are presented as %RSD over three validation days (*n* = 9).
**Data S6:** OLAE storage trial data at −20°c (up to 189 days) for analytes of interest. Results are presented as average ± SD (*n* = 3).
**Data S7:** OLAE storage trial data at 24°c (up to 189 days) for analytes of interest. Results are presented as average ± SD (*n* = 3).
**Data S8:** OLAE storage trial data at 40°c (up to 189 days) for analytes of interest. Results are presented as average ± SD (*n* = 3).

## Data Availability

The data that supports the findings of this study are available in the Data S1–S8 of this article.
