# Supplementary material for: Using Olive Leaves to Enrich Olive Oil With Oleuropein Aglycone
Source: Food Sci Nutr. 2026 Oct 23;14(10):e72421. doi: 10.1002/fsn3.72421 (PMC13694744; doi:10.1002/fsn3.72421)
Supplement: Supplementary file 1 [file FSN3-14-e72421-s001.docx]

**Supporting Information**

**Using olive leaves to enrich olive oil with oleuropein aglycone**

Jade te Bogt^1^, David Speed^2^, Ralf C. Schlothauer^3^, Jed W. Fahey^4,5^, Megan N. C. Grainger^1^*

^1^ School of Science, Division of STEM, The University of Waikato, Hamilton, New Zealand

^2^ ALS Food & Environmental NZ, formerly Analytica Laboratories, Hamilton, New Zealand

^3^ Whio Innovations Ltd

^4^ Departments of Medicine, Physiology, Pharmacology & Therapeutics, and Psychiatry & Behavioral Sciences, School of Medicine, Johns Hopkins University, Baltimore, Maryland, USA

^5^ Institute of Medicine, University of Maine, Orono, Maine, USA

*megan.grainger@waikato.ac.nz; +64 7 337 9621

Jade te Bogt - Current address: ALS Food & Environmental NZ, Hamilton, New Zealand

**Method validation for olive oil**

**Supporting Information S1 Limits of detection and quantification for target analytes when analysed by LC-MS/MS.**

| **Analyte** | **LOD** | | **LOQ** | |
| --- | --- | --- | --- | --- |
|  | **µg/L** | **mg/kg** | **µg/L** | **mg/kg** |
| Oleuropein | 1.82 | 0.14 | 6.07 | 0.47 |
| Ligstroside | 1.48 | 0.13 | 4.92 | 0.43 |
| Oleuropein aglycone | 2.17 | 0.10 | 7.22 | 0.34 |
| Oleacein | 0.99 | 0.04 | 3.31 | 0.13 |
| Oleocanthal | 3.67 | 0.16 | 12.24 | 0.53 |
| Hydroxytyrosol | 2.64 | 0.16 | 8.81 | 0.55 |
| Tyrosol | 5.16 | 0.31 | 17.20 | 1.03 |
| Elenolic acid | 5.78 | 0.35 | 19.26 | 1.18 |

****Supporting Information S2**** ****Endogenous, high**** and ****low spike sample concentrations (µg/L) and spike recoveries for olive oil (OO) (%). Results are presented as the average ± SD (*n* = 9).****

| **Analyte** | **Endogenous concentration** | **Concentration (µg/L)** | | **% Recovery** | | **Concentration (µg/L)** | | **% Recovery** | |
| --- | --- | --- | --- | --- | --- | --- | --- | --- | --- |
|  |  | **20 µg/L spike^◊^** | | | | **50 µg/L spike^◊^** | | | |
| **OO 1 (µg/L)** | | | | | | | | | |
| Oleuropein | <LOD | 19.4 ± 0.6 | | 97 ± 3 | | 51.4 ± 4.8 | | 103 ± 7 | |
| Ligstroside | <LOD | 20.6 ± 0.5 | | 103 ± 2 | | 53.7 ± 5.2 | | 107 ± 8 | |
| Oleuropein aglycone | 19.6 ± 0.7 | 39.7 ± 0.6 | | 100 ± 1 | | 72.2 ± 3.8 | | 105 ± 5 | |
| Oleacein | 10.0 ± 0.3 | 32.0 ± 0.5 | | 110 ± 3 | | 65.2 ± 5.6 | | 110 ± 8 | |
| Oleocanthal | 34.3 ± 1.2 | 54.6 ± 1.6 | | 102 ± 7 | | 85.2 ± 5.6 | | 102 ± 5 | |
| Hydroxytyrosol | 4.3* ± 0.5 | 24.6 ± 0.8 | | 102 ± 1 | | 55.7 ± 5.4 | | 103 ± 5 | |
| Tyrosol | <LOD | 23.7 ± 1.7 | | 110 ± 1 | | 55.8 ± 5.2 | | 106 ± 6 | |
| Elenolic acid | 38.1 ± 1.6 | 59.3 ± 2.2 | | 106 ± 7 | | 91.5 ± 6.2 | | 107 ± 13 | |
| **OO 2 (µg/L)** | | | | | | | | | |
| Oleuropein | <LOD | | 19.4 ± 0.6 | | 97 ± 3 | | 52.1 ± 1.6 | | 104 ± 3 |
| Ligstroside | <LOD | | 19.9 ± 0.5 | | 100 ± 2 | | 53.5 ± 1.8 | | 107 ± 3 |
| Oleuropein aglycone | 21.9 ± 1.0 | | 42.9 ± 0.8 | | 105 ± 2 | | 75.3 ± 1.8 | | 107 ± 4 |
| Oleacein | 56.9 ± 1.8 | | 78.6 ± 1.8 | | 109 ± 5 | | 110.1 ± 2.4 | | 106 ± 5 |
| Oleocanthal | 196.9 ± 7.2 | | 216.7 ± 4.5 | | 104 ± 28 | | 245.7 ± 4.0 | | 100 ± 11 |
| Hydroxytyrosol | 4.1* ± 0.4 | | 24.6 ± 0.8 | | 102 ± 2 | | 57.8 ± 3.1 | | 107 ± 5 |
| Tyrosol | <LOD | | 24.0 ± 2.0 | | 108 ± 6 | | 55.9 ± 3.5 | | 107 ± 3 |
| Elenolic acid | 30.4 ± 1.9 | | 52.1 ± 2.3 | | 108 ± 4 | | 85.3 ± 4.6 | | 110 ± 7 |

****Supporting Information S3 OO 1 intra- and inter-day %RSDs for endogenous, low and high spike samples. Intra-day results (*n* = 3) are presented as the range of %RSDs from each validation day. The inter-day precision is presented as %RSD over three days (*n* = 9).****

| **Analyte** | **OO 1 intra-day precision (%RSD, *n* = 3)** | | |
| --- | --- | --- | --- |
|  | **Endogenous** | **Low spike** | **High spike** |
| Oleuropein | n/a^◊^ | 2.01-2.91 | 2.92-8.61 |
| Ligstroside | n/a^◊^ | 0.45-2.50 | 1.71-6.84 |
| Oleuropein aglycone | 0.47-2.20 | 0.86-1.16 | 1.68-4.38 |
| Oleacein | 1.33-2.89 | 0.86-1.72 | 1.12-8.81 |
| Oleocanthal | 0.37-3.45 | 1.20-1.65 | 1.47-7.60 |
| Hydroxytyrosol | n/a^◊^ | 1.99-3.84 | 1.19-11.43 |
| Tyrosol | n/a^◊^ | 2.17-7.10 | 2.97-6.65 |
| Elenolic acid | 0.10-2.99 | 1.94-3.93 | 1.28-4.09 |
|  | **Inter-day precision (%RSD, *n* = 9)** | | |
| Oleuropein | n/a^◊^ | 3.44 | 9.46 |
| Ligstroside | n/a^◊^ | 2.93 | 9.96 |
| Oleuropein aglycone | 2.95 | 1.09 | 5.29 |
| Oleocanthal | 2.60 | 2.50 | 6.30 |
| Oleacein | 2.25 | 2.30 | 8.83 |
| Hydroxytyrosol | n/a^◊^ | 3.70 | 9.62 |
| Tyrosol | n/a^◊^ | 7.24 | 9.38 |
| Elenolic acid | 4.90 | 3.62 | 6.82 |

*^◊^n/a – not applicable: values were <LOD so no %RSDs could be determined.*

****Supporting Information S4 OO 2 intra- and inter-day %RSDs for endogenous, low spike and higher spike samples. Intra-day results (*n* = 3) are presented as the range of %RSDs from each validation day. The inter-day precision is presented as %RSD over three days (*n* = 9).****

| **Analyte** | **OO 2 intra-day precision (%RSD, *n* = 3)** | | |
| --- | --- | --- | --- |
|  | **Endogenous** | **Low spike** | **High spike** |
| Oleuropein | n/a^◊^ | 0.86-1.52 | 1.70-2.61 |
| Ligstroside | n/a^◊^ | 0.71-1.80 | 0.67-1.99 |
| Oleuropein aglycone | 1.11-3.69 | 1.05-1.62 | 1.07-2.07 |
| Oleacein | 0.34-1.97 | 0.24-1.83 | 0.18-0.95 |
| Oleocanthal | 1.06-2.40 | 0.62-1.59 | 1.37-1.93 |
| Hydroxytyrosol | n/a^◊^ | 1.83-2.62 | 1.33-2.91 |
| Tyrosol | n/a^◊^ | 2.53-5.33 | 0.37-4.87 |
| Elenolic acid | 0.50-6.57 | 1.36-5.47 | 0.18-3.94 |
|  | **Inter-day precision (%RSD, *n* = 9)** | | |
| Oleuropein | n/a^◊^ | 4.02 | 3.25 |
| Ligstroside | n/a^◊^ | 3.57 | 3.24 |
| Oleuropein aglycone | 3.72 | 1.62 | 2.24 |
| Oleacein | 2.41 | 1.57 | 2.31 |
| Oleocanthal | 3.00 | 1.30 | 1.81 |
| Hydroxytyrosol | n/a^◊^ | 3.24 | 5.32 |
| Tyrosol | n/a^◊^ | 8.02 | 6.36 |
| Elenolic acid | 6.47 | 4.44 | 5.30 |

*^◊^n/a – not applicable: values were <LOD so no %RSDs could be determined.*

**Method validation for olive leaf aqueous extract (OLAE)**

****Supporting Information S5**** Spike recoveries, i****ntra-day and inter-day precision ranges for the target analytes using 80 µg/L spiked OLAE data. Spike results are presented as the average ± SD (*n* = 9). Intra-day results are presented as the range of %RSDs from each validation day (*n* = 3). Inter-day results are presented as %RSD over three validation days (*n* = 9).****

| **Analyte** | **Spike recovery (%) (*n* = 9)** | **Intra-day precision**  **(%RSD, *n* = 3)** | **Inter-day precision**  **(%RSD, *n* = 9)** |
| --- | --- | --- | --- |
| Oleuropein | 91 ± 4 | 0.53-2.94 | 4.95 |
| Ligstroside | 93 ± 2 | 1.78-5.43 | 3.73 |
| Oleuropein aglycone | 80 ± 11 | 3.26-4.45 | 13.21 |
| Oleacein | 99 ± 7 | 0.38-3.25 | 7.21 |
| Oleocanthal | 94 ± 4 | 1.41-2.36 | 6.06 |
| Hydroxytyrosol | 105 ± 4 | 1.89-5.25 | 5.97 |
| Tyrosol | 98 ± 2 | 1.20-2.46 | 3.25 |
| Elenolic acid | 112 ± 9 | 2.52-7.18 | 9.33 |

**Supporting Information S6 OLAE storage trial data at –20 °C (up to 189 days) for analytes of interest. Results are presented as average ± SD (*n* = 3).**

| **Day** | **Temp (°C)** | **Oleuropein** | **Oleocanthal** | **Oleacein** | **Hydroxytyrosol** | **Tyrosol** | **Oleuropein aglycone** | **Ligstroside** | **Elenolic acid** |
| --- | --- | --- | --- | --- | --- | --- | --- | --- | --- |
|  |  | **(mg/kg)** | | | | | | | |
| 0 | -20 | 343 ± 3 | 15.9 ± 0.1 | 221 ± 9 | 981 ± 40 | 25.9 ± 1.7 | 13.5 ± 0.5 | 63 ± 11 | 475 ± 49 |
| 29 | -20 | 373 ± 6 | 16.0 ± 0.1 | 196 ± 3 | 1034 ± 37 | 31.9 ± 0.5 | 13.8 ± 0.8 | 77 ± 2 | 547 ± 5 |
| 41 | -20 | 358 ± 17 | 16.1 ± 0.4 | 181 ± 1 | 994 ± 5 | 37.8 ± 1.0 | 12.9 ± 0.3 | 74 ± 1 | 482 ± 9 |
| 50 | -20 | 347 ± 11 | 16.4 ± 1.0 | 183 ± 1 | 1030 ± 3 | 26.3 ± 0.1 | 14.1 ± 0.6 | 73 ± 1 | 460 ± 16 |
| 56 | -20 | 353 ± 7 | 15.1 ± 1.2 | 183 ± 7 | 1059 ± 14 | 27.1 ± 1.1 | 14.8 ± 0.6 | 75 ± 4 | 458 ± 9 |
| 79 | -20 | 342 ± 14 | 17.6 ± 0.1 | 152 ± 0.2 | 968 ± 17 | 34.7 ± 2.5 | 16.3 ±0.6 | 74 ± 3 | 400 ± 8 |
| 100 | -20 | 351 ± 13 | 14.7 ± 0.1 | 157 ± 9 | 981 ± 40 | 26.7 ± 0.1 | 16.5 ± 1.0 | 75 ± 3 | 422 ± 11 |
| 112 | -20 | 355 ± 22 | 15.0 ± 1.0 | 176 ± 3 | 992 ± 60 | 31.6 ± 0.6 | 13.8 ± 0.6 | 67 ± 1 | 391 ± 22 |
| 122 | -20 | 366 ± 10 | 14.6 ± 0.1 | 171 ± 5 | 1212 ± 8 | 27.7 ± 0.8 | 16.7 ± 0.5 | 78 ± 4 | 433 ± 14 |
| 129 | -20 | 352 ± 7 | 15.1 ± 0.2 | 164 ± 7 | 1006 ± 8 | 27.4 ± 0.6 | 12.6 ± 1.1 | 72 ± 1 | 504 ± 11 |
| 141 | -20 | 338 ± 8 | 13.7 ± 0.5 | 142 ± 12 | 933 ± 39 | 21.9 ± 0.8 | 13.3 ± 0.002 | 63 ± 3 | 431 ± 37 |
| 150 | -20 | 339 ± 12 | 13.8 ± 1.0 | 145 ± 3 | 957 ± 18 | 27.1 ± 0.6 | 13.2 ± 0.3 | 72 ± 1 | 475 ± 13 |
| 156 | -20 | 353 ± 9 | 13.0 ± 0.7 | 161 ± 4 | 1030 ± 18 | 25.5 ± 1.1 | 12.6 ± 0.6 | 73 ± 4 | 478 ± 11 |
| 164 | -20 | 338 ± 10 | 13.2 ± 0.2 | 153 ± 4 | 955 ± 37 | 24.5 ± 0.8 | 12.7 ± 0.2 | 74 ± 2 | 451 ± 11 |
| 174 | -20 | 348 ± 14 | 14.1 ± 0.2 | 153 ±6 | 911 ± 18 | 30.7 ± 1.5 | 10.2 ± 2.3 | 79 ± 4 | 492 ± 27 |
| 181 | -20 | 363 ± 10 | 14.1 ± 0.3 | 158 ± 9 | 1005 ± 46 | 26.8 ± 0.5 | 13.4 ± 0.2 | 76 ± 3 | 482 ± 31 |
| 189 | -20 | 355 ± 5 | 13.9 ± 0.1 | 152 ± 5 | 971 ± 41 | 29.1 ± 0.4 | 13.3 ± 0.1 | 76 ± 2 | 470 ± 15 |

**Supporting Information S7 OLAE storage trial data at 24 °C (up to 189 days) for analytes of interest. Results are presented as average ± SD (*n* = 3).**

| **Day** | **Temp (°C)** | **Oleuropein** | **Oleocanthal** | **Oleacein** | **Hydroxytyrosol** | **Tyrosol** | **Oleuropein aglycone** | **Ligstroside** | **Elenolic acid** |
| --- | --- | --- | --- | --- | --- | --- | --- | --- | --- |
|  |  | **(mg/kg)** | | | | | | | |
| 0 | 24 | 343 ± 3 | 15.9 ± 0.1 | 221 ± 9 | 981 ± 40 | 25.9 ± 1.7 | 13.5 ± 0.5 | 62.5 ± 11.4 | 475 ± 49 |
| 29 | 24 | 348 ± 13 | 13.8 ± 0.7 | 222 ± 1 | 927 ± 9 | 31.7 ± 1.1 | 10.2 ± 0.9 | 77.9 ± 2.4 | 361 ± 15 |
| 41 | 24 | 317 ± 2 | 16.5 ± 0.3 | 209 ± 2 | 853 ± 16 | 32.6 ± 0.9 | 7.8 ± 0.9 | 72.0 ± 0.4 | 286 ± 0.8 |
| 50 | 24 | 303 ± 10 | 14.6 ± 0.1 | 221 ± 2 | 878 ± 7 | 31.8 ± 2.3 | 8.4 ± 0.1 | 70.4 ± 1.0 | 281 ± 0.5 |
| 56 | 24 | 295 ± 9 | 17.6 ± 1.5 | 222 ± 3 | 877 ± 2 | 28.8 ± 0.7 | 8.9 ± 0.1 | 70.9 ± 2.4 | 254 ± 3 |
| 79 | 24 | 317 ± 3 | 20.4 ± 0.2 | 223 ± 2 | 874 ± 19 | 33.8 ± 1.8 | 9.1 ± 0.5 | 72.9 ± 0.03 | 246 ± 3 |
| 100 | 24 | 327 ± 10 | 17.6 ± 0.1 | 239 ± 1 | 890 ± 18 | 34.3 ± 1.9 | 8.2 ± 0.1 | 76.0 ± 3.2 | 246 ± 1.5 |
| 112 | 24 | 327 ± 14 | 20.5 ± 1.5 | 259 ± 11 | 942 ± 29 | 35.3 ± 1.9 | 8.5 ± 0.3 | 74.4 ± 3.5 | 308 ± 3 |
| 122 | 24 | 305 ± 10 | 18.4 ± 0.1 | 261 ± 9 | 1021 ± 14 | 36.7 ± 5.9 | 9.5 ± 0.5 | 80.1 ± 5.9 | 247 ± 3 |
| 129 | 24 | 278 ± 8 | 18.5 ± 0.3 | 239 ± 6 | 793 ± 1 | 34.6 ± 5.0 | 7.8 ± 0.8 | 70.2 ± 1.5 | 271 ± 4 |
| 141 | 24 | 285 ± 6 | 17.7 ± 0.7 | 238 ± 13 | 855 ± 25 | 34.7 ± 1.1 | 7.5 ± 0.1 | 72.1 ± 2.7 | 290 ± 16 |
| 150 | 24 | 257 ± 10 | 18.2 ± 0.3 | 216 ± 4 | 733 ± 17 | 31.0 ± 0.5 | 6.9 ±0.1 | 69.6 ± 0.9 | 262 ± 5 |
| 156 | 24 | 285 ± 5 | 20.6 ± 0.1 | 252 ± 4 | 811 ± 30 | 36.7 ± 3.2 | 6.5 ± 0.5 | 74.6 ± 3.9 | 278 ± 11 |
| 164 | 24 | 268 ± 8 | 19.2 ± 1.5 | 235 ± 5 | 776 ± 24 | 40.0 ± 3.0 | 7.1 ± 0.1 | 75.0 ± 1.3 | 267 ± 12 |
| 174 | 24 | 265 ± 7 | 18.7 ± 0.2 | 237 ± 15 | 785 ± 42 | 32.5 ± 0.7 | 6.3 ± 0.6 | 77.0 ± 3.1 | 285 ± 10 |
| 181 | 24 | 264 ± 5 | 19.3 ± 0.2 | 228 ± 13 | 754 ± 37 | 37.5 ± 3.9 | 6.7 ± 0.2 | 75.0 ± 1.7 | 272 ± 15 |
| 189 | 24 | 255 ± 7 | 18.7 ± 0.5 | 226 ± 7 | 709 ± 16 | 39.4 ± 1.7 | 7.1 ± 0.2 | 72.8 ± 1.8 | 285 ± 7 |

**Supporting Information S8 OLAE storage trial data at 40 °C (up to 189 days) for analytes of interest. Results are presented as average ± SD (*n* = 3).**

| **Day** | **Temp (°C)** | **Oleuropein** | **Oleocanthal** | **Oleacein** | **Hydroxytyrosol** | **Tyrosol** | **Oleuropein aglycone** | **Ligstroside** | **Elenolic acid** |
| --- | --- | --- | --- | --- | --- | --- | --- | --- | --- |
|  |  | **(mg/kg)** | | | | | | | |
| 0 | 40 | 343 ± 3 | 15.9 ± 0.1 | 221 ± 9 | 981 ± 40 | 25.9 ± 1.7 | 13.5 ± 0.5 | 70.8 ± 11.4 | 475 ± 49 |
| 29 | 40 | 294 ± 13 | 23.5 ± 0.9 | 387 ± 2 | 904 ± 14 | 41.9 ± 2.0 | 15.4 ± 1.0 | 67.4 ± 1.7 | 437 ± 29 |
| 41 | 40 | 267 ± 13 | 23.3 ± 1.0 | 356 ± 5 | 988 ± 2 | 37.2 ± 3.2 | 17.4 ± 0.7 | 64.7 ± 0.9 | 459 ± 18 |
| 50 | 40 | 244 ± 6 | 23.2 ± 1.1 | 339 ± 11 | 959 ± 23 | 38.0 ± 0.3 | 19.3 ± 0.9 | 59.8 ± 0.7 | 457 ± 5 |
| 56 | 40 | 232 ± 3 | 21.0 ± 0.2 | 312 ± 13 | 948 ± 23 | 39.5 ± 0.2 | 19.5 ± 0.7 | 60.2 ± 1.0 | 456 ± 20 |
| 79 | 40 | 189 ± 8 | 19.9 ± 0.5 | 217 ± 13 | 903 ± 13 | 46.5 ± 5.3 | 26.0 ± 0.4 | 48.9 ± 2.5 | 473 ± 2 |
| 100 | 40 | 168 ±7 | 13.2 ± 0.1 | 162 ± 8 | 925 ± 32 | 43.0 ± 2.0 | 23.3 ± 0.7 | 45.2 ± 0.7 | 536 ± 11 |
| 112 | 40 | 155 ± 2 | 13.6 ± 0.1 | 152 ± 5 | 1001 ± 29 | 46.2 ±3.2 | 22.8 ± 1.1 | 40.5 ± 1.9 | 694 ± 11 |
| 122 | 40 | 133 ± 3 | 12.2 ± 0.4 | 138 ± 4 | 1080 ± 7 | 44.0 ± 0.6 | 23.7 ± 0.3 | 42.2 ± 2.0 | 543 ± 10 |
| 129 | 40 | 110 ± 5 | 8.5 ± 0.1 | 108 ± 4 | 848 ± 25 | 47.4 ± 0.3 | 16.6 ± 1.0 | 33.9 ± 0.3 | 596 ± 7 |
| 141 | 40 | 103 ± 4 | 9.0 ± 0.2 | 97 ± 5 | 912 ± 44 | 46.9 ± 1.1 | 18.5 ± 0.7 | 32.1 ± 1.9 | 659 ± 38 |
| 150 | 40 | 80 ± 4 | 7.8 ± 0.5 | 77 ± 3 | 756 ± 36 | 44.5 ± 3.2 | 16.3 ±0.4 | 28.4 ± 1.0 | 635 ± 32 |
| 156 | 40 | 80 ± 4 | 7.8 ± 0.1 | 81 ± 1 | 839 ± 13 | 49.7 ± 0.2 | 16.4 ± 0.7 | 28.1 ± 4.2 | 700 ± 19 |
| 164 | 40 | 69 ± 2 | 7.0 ± 0.2 | 68 ± 3 | 783 ± 22 | 48.7 ± 2.0 | 15.2 ± 0.4 | 26.4 ± 1.1 | 650 ± 38 |
| 174 | 40 | 64 ± 1 | 6.4 ± 0.1 | 62 ± 2 | 763 ± 27 | 53.1 ± 4.3 | 12.6 ± 1.4 | 26.1 ± 0.4 | 671 ± 44 |
| 181 | 40 | 56 ± 1 | 5.5 ± 0.1 | 52 ± 3 | 767 ± 55 | 59.7 ± 0.5 | 13.2 ± 0.3 | 21.8 ± 1.0 | 611 ± 26 |
| 189 | 40 | 48 ± 2 | 5.0 ± 0.3 | 45 ± 1 | 722 ± 37 | 54.0 ± 0.7 | 12.1 ± 0.4 | 19.7 ± 0.8 | 667 ± 33 |
